# Resolving Ambiguities in the LF/HF Ratio: LF-HF Scatter Plots for the Categorization of Mental and Physical Stress from HRV

**DOI:** 10.3389/fphys.2017.00360

**Published:** 2017-06-14

**Authors:** Wilhelm von Rosenberg, Theerasak Chanwimalueang, Tricia Adjei, Usman Jaffer, Valentin Goverdovsky, Danilo P. Mandic

**Affiliations:** ^1^Department of Electrical and Electronic Engineering, Imperial College LondonLondon, United Kingdom; ^2^Department of Vascular Surgery, Imperial College School of Medicine, Hammersmith HospitalLondon, United Kingdom

**Keywords:** heart rate variability (HRV), stress categorization, mental stress, physical stress, LF/HF-ratio, wearable ECG

## Abstract

It is generally accepted that the activities of the autonomic nervous system (ANS), which consists of the sympathetic (SNS) and parasympathetic nervous systems (PNS), are reflected in the low- (LF) and high-frequency (HF) bands in heart rate variability (HRV)—while, not without some controversy, the ratio of the powers in those frequency bands, the so called LF-HF ratio (LF/HF), has been used to quantify the degree of sympathovagal balance. Indeed, recent studies demonstrate that, in general: (i) sympathovagal balance cannot be accurately measured via the ratio of the LF- and HF- power bands; and (ii) the correspondence between the LF/HF ratio and the psychological and physiological state of a person is not unique. Since the standard LF/HF ratio provides only a single degree of freedom for the analysis of this 2D phenomenon, we propose a joint treatment of the LF and HF powers in HRV within a two-dimensional representation framework, thus providing the required degrees of freedom. By virtue of the proposed 2D representation, the restrictive assumption of the linear dependence between the activity of the autonomic nervous system (ANS) and the LF-HF frequency band powers is demonstrated to become unnecessary. The proposed analysis framework also opens up completely new possibilities for a more comprehensive and rigorous examination of HRV in relation to physical and mental states of an individual, and makes possible the categorization of different stress states based on HRV. In addition, based on instantaneous amplitudes of Hilbert-transformed LF- and HF-bands, a novel approach to estimate the markers of stress in HRV is proposed and is shown to improve the robustness to artifacts and irregularities, critical issues in real-world recordings. The proposed approach for resolving the ambiguities in the standard LF/HF-ratio analyses is verified over a number of real-world stress-invoking scenarios.

## 1. Introduction

The analysis of heart rate variability (HRV) has become a standard in the estimation of the state of body and mind in humans, with multiple indices derived from HRV being routinely used in the analysis. The HRV is a time series of the variation of the heart rate over time and is obtained by identifying the QRS-complexes, the most pronounced feature in the cardiac-cycle, and calculating the difference in time between two consecutive occurrences of QRS-complexes, the so called normal-to-normal interval (NNI). The NNI time series yields a much more informative basis for further stress analysis than the raw electrocardiogram (ECG). The heart contains pacemaker cells which spontaneously depolarize when their membrane potential reaches a certain threshold (Reisner et al., 2006). The influx speed of ions which depolarize the cells is partially driven by the autonomic nervous system (ANS), whereby the sympathetic nervous system (SNS) increases the conductivity of the cell membrane, leading to shorter intervals between two heart beats and hence a higher heart rate, while the parasympathetic nervous system (PNS) has the opposite effect, leading to a lower heart rate (Shaffer et al., 2014). Therefore, the HRV provides an insight into both the two nervous systems (SNS and PNS) and their interaction, the so called sympathovagal balance.

The features of the NNI time series relevant for stress assessment are typically obtained in the time and frequency domains, and more recently through parameters which reflect structural complexity of the NNI time series (complexity science) (Williamon et al., 2013). These measurements, methods, and nomenclature for ECG and HRV have been standardized by a task force within The European Society for Cardiology and the North American Society of Pacing and Electrophysiology (Malik et al., 1996), and their recommendations have been generally accepted in both research and clinical practice.

Algorithms for the calculation of HRV-parameters are typically applied to sliding time windows (or epochs) of the NNI time series. The time domain characteristics include (among others): (i) standard deviation of NN-intervals (SDNN), (ii) square root of the mean of the sum of the squares of differences between successive NN-intervals (RMSSD), and (iii) proportion of the number of NN-interval differences of successive NN-intervals which are greater than (Malik et al., 1996). However, importantly, these measures do not necessarily indicate whether a change in HRV had been caused by the SNS or the PNS. The same issue affects complexity science measures—the structural complexity of the NNI time series can decrease because of a *clear dominance of a deterministic component* in either nervous system. Frequency domain analyses are better equipped to discriminate between the contributions of the SNS and PNS, as they manifest themselves in two non-overlapping frequency bands. Empirical evidence suggests that the activity of the SNS influences the low frequency band (LF) of the HRV, from 0.04 to 0.15 Hz, while the PNS is predominantly reflected in the high frequency band (HF), from 0.15 to 0.4 Hz, and also possibly in a proportion of LF (Malik et al., 1996). To differentiate between the frequency bands in HRV and the total signal powers contained in these bands, we have adopted a notation whereby a subscript “p” designates the power in a frequency band of interest. Having in mind that an increased power in the low frequency band (LF_p_) implies a more dominant activity of the SNS while an increased power in the high frequency band (HF_p_) indicates a stronger influence of the PNS, Pagani et al. (1986) proposed to combine LF_p_ and HF_p_ into the low-to-high frequency ratio (LF/HF) as an index for the sympathovagal balance between the two nervous systems. The authors also studied the correlation between sympathetic nerve activity in muscles and the information in the frequency bands of HRV in humans (Pagani et al., 1997). The LF_p_, HF_p_, and LF/HF have been subsequently adopted as markers of stress in a number of studies (Malliani et al., 1991, 1997; Montano et al., 1994). More recently, it was realized that the LF/HF is in general not a reliable metric; for example, a result contrary to that theory was described by Arai et al. (1989) for physical stress, where the LF_p_ decreased for increasing exercise levels.

The LF/HF has also received some criticism as a measure of cognitive and physical aspects of stress. Eckberg (1997) scrutinized the relationship between SNS and LF_p_ and between PNS and HF_p_ and found remarkable inconsistencies, a finding which has itself received much counter-criticism. In a comprehensive study by Billman (2013b), it was conclusively shown that sympathovagal balance cannot be quantified by a single number, the LF/HF, which assumes a simplistic linear relationship between the activity of the nervous systems and the frequency bands.

In this study, we deviate from the traditional interpretation of the sympathovagal balance via the LF/HF and propose a joint two-dimensional representation of the information in the low- and high-frequency HRV bands. The so obtained additional degree of freedom enables a rigorous categorization of stress, without resorting to a restrictive linear relationship between the underlying stress level and the LF_p_ and HF_p_, or a reciprocal relationship between LF_p_ and HF_p_. This also allows for more rigor in the examination of HRV and reduces ambiguities in stress categorization, as demonstrated over a quantitative comparison of the effect of standardized mental and physical stress on the HRV of 10 subjects, and for a range of scenarios. Finally, for enhanced robustness to ectopic beats and other abrupt disturbances to the NNI time series, a new metric for the estimation of the activity in the LF and HF is proposed, which benefits from the enhanced resolution of instantaneous amplitudes of the LF and HF time series obtained through the Hilbert transform.

## 2. Methods

### 2.1. Standard stress analysis

In all experiments, NNI time series were created through the extraction of the timings of R-waves from one or multiple ECG-channels using our state-of-the-art software for the detection of QRS complexes (Chanwimalueang et al., 2015). For reliable estimation, the algorithms for the calculation of stress parameters are usually applied to time windows with a length of at least 5 min. This ensures that sufficiently many oscillations of the lowest frequency of interest, which corresponds to the lower cut-off of LF (0.04 Hz $\hat{=}$ 25 s), are present in every time window under consideration; in our case, this was 12 cycles for 300 s time windows and 10 cycles for 250 s time windows. The standard analyses treat the data either as a whole epoch of interest or segment the data into overlapping sliding time windows of 5 min in duration and with a 10 s sliding increment. Subsequently, various features in the time or frequency domains are analyzed in response to different stress events in a recording. Three popular characterization parameters in the frequency domain are the previously mentioned LF_p_ and HF_p_ which—in a simplified view—are respectively thought to roughly indicate the activity of the SNS and PNS, and their ratio (LF_p_/HF_p_). A large LF_p_/HF_p_ value assigns a high stress level to a time window under consideration, and indicates a strong influence of the SNS, a weak influence of the PNS, or both. We should reiterate that this simplified approach has been scrutinized in recent years.

### 2.2. Two-dimensional assessment of stress parameters

Mathematically, the exploitation of the full available information in SNS and PNS requires two degrees of freedom, yet current metrics, such as the LF/HF ratio, are one-dimensional and therefore cannot fully address the impact of the SNS and PNS on the HRV. For example, a high SDNN can be caused by an increased activity of either the SNS, the PNS, or both. The rationale for the ratio of LF_p_ and HF_p_ as a universal stress metric is that a high LF_p_ indicates high stress (an increase in the activity of the SNS) while a high HF_p_ indicates low stress (increased activity of the PNS). To further support the finding that the LF/HF is neither a unique or optimal univariate metric, we next introduce a class of arbitrary (univariate) LF_p_-HF_p_ relationships which fulfil the requirements for a stress metric, that is: (i) to increase with an increase in LF_p_ and a decrease in HF_p_, and (ii) to decrease with a decrease in LF_p_ and an increase in HF_p_. Four such alternative metrics (*P*_*i*_, *i* = 2, 3, 4, 5) to quantify the sympathovagal balance are given below Equations (1b–1e), against the existing reference LF_p_/HF_p_ relationship in *P*_1_:

(1) $$\begin{array}{l} a)\;P_{1}=\frac{LF_{p}}{HF_{p}}\\ b)\;P_{2}=b_{1}\cdot \frac{LF_{p}^{2}}{HF_{p}}+b_{2}\\ c)\;P_{3}=c_{1}\cdot LF_{p}-c_{2}\cdot HF_{p}+c_{3}\\ d)\;P_{4}=d_{1}\cdot LF_{p}^{2}-d_{2}\cdot HF_{p}+d_{3}\\ e)\;P_{5}=e_{1}\cdot \frac{LF_{p}}{HF_{p}^{2}}+e_{2} \end{array}$$

**Figure 1:**
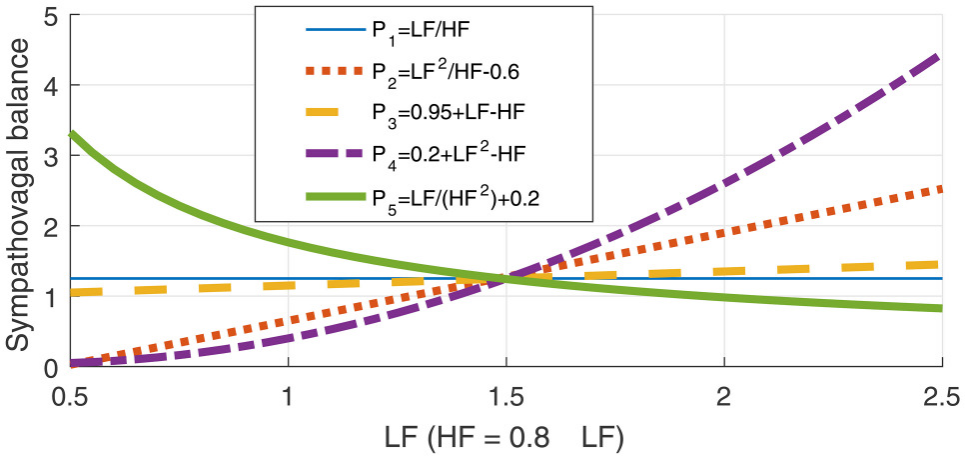
Illustration of the ambiguity in the LF-HF ratio through a class of possible relationships between the low- (LF) and high-frequency bands (HF) in heart rate variability (HRV), given in Equation (1). The metrics *P*_1_ to *P*_5_ represent possible sympathovagal balance relationships and are designed so that every metric *P*_*i*_ increases with the activity of the sympathetic nervous system (SNS), and decreases with an increase in the activity of the parasympathetic nervous system (PNS). However, observe that for a constant LF_p_/HF_p_ ratio, which is believed to indicate a constant sympathovagal balance, the curves for various *P*_*i*_, *i* = 1, 2, …, 5 in Equation (1) behave quite differently, which demonstrates the inadequacy of any 1D metrics for the assessment of stress.

The parameters *b*, *c*, *d*, and *e* in Equation (1) are user-defined constants. According to the standard LF-HF theory (Equation 1a), the balance between the SNS and PNS is reflected in a fixed LF_p_/HF_p_ ratio, independent of the absolute values of LF_p_ and HF_p_. However, according to the requirements in (i) and (ii) above, Equations (1b–1e) are also valid metrics for the sympathovagal balance, yet these each yield different results and have different interpretations. This is further demonstrated in Figure 1, which shows the metrics *P*_1_ to *P*_5_, generated for a varying LF_p_ and an arbitrary but realistic LF_p_/HF_p_ value of 1.25. Notice that while the assumptions (i) and (ii) regarding LF_p_ and HF_p_ and their effects on the metrics *P*_*i*_ are fulfilled for all the curves, for a constant LF_p_/HF_p_ = 1.25 ⇔ HF_p_ = 0.8·LF_p_, the curves *P*_2_ to *P*_5_ behave differently—from monotonically increasing (*P*_2_, *P*_3_, *P*_4_) to monotonically decreasing (*P*_5_). This further exemplifies the ambiguities inherent to the one-dimensional class of stress metrics in Equation (1), and their limitations in the analysis of the multifaceted stress phenomenon.

Therefore, the use of the traditional LF/HF—or any other formula in Equation (1)—to unify all LF-HF pairs which exhibit a certain relationship (e.g., the same LF-HF-quotient or LF-HF-difference) into a single value induces a loss of one degree of freedom. This motivates us to propose a new metric, in the form of a 2D scatter plot of LF vs. HF, which maintains both existing degrees of freedom and thus utilizes all available information in LF and HF. In addition, this analysis framework allows for different psychological and physiological scenarios to be considered within the same 2D diagram, therefore making possible both their joint analysis and enhanced discrimination. (Alternatively, the proposed framework also enables the consideration of 3D scatter plots, e.g., with HF on the x-axis, LF on the y-axis, and the time on the z-axis, in order to examine the evolution of stress levels in time.) Another important advantage of the proposed LF-HF scatter diagram is the inherent uncoupling of the LF/HF quotient into its LF and HF components, which enables the discrimination between LF/HF pairs with identical ratios, a critical problem with current metrics, which do not posses this additional degree of freedom.

**Figure 2:**
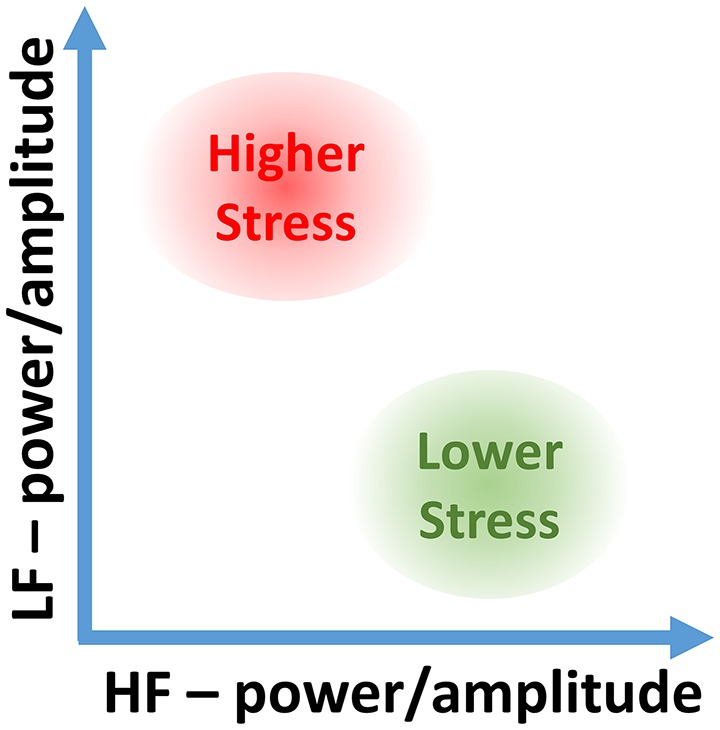
Stress categorization in the proposed 2D LF-HF diagram: interpretation of characteristic areas in the two-dimensional scatter plot of the low- (LF) and high-frequency (HF) power or amplitude of heart rate variability (HRV).

The proposed 2D analysis framework is also amenable to a subsequent use of machine learning; for example, in the LF-HF scatter plots, the characteristic levels of psychological and physiological arousal now occupy their own distinctive areas. Two such representative areas, when both high LF_p_ and low HF_p_ activities indicate high stress, and vice versa for low stress, are shown in Figure 2.

### 2.3. Instantaneous amplitude as a stress metric

**Figure 3:**
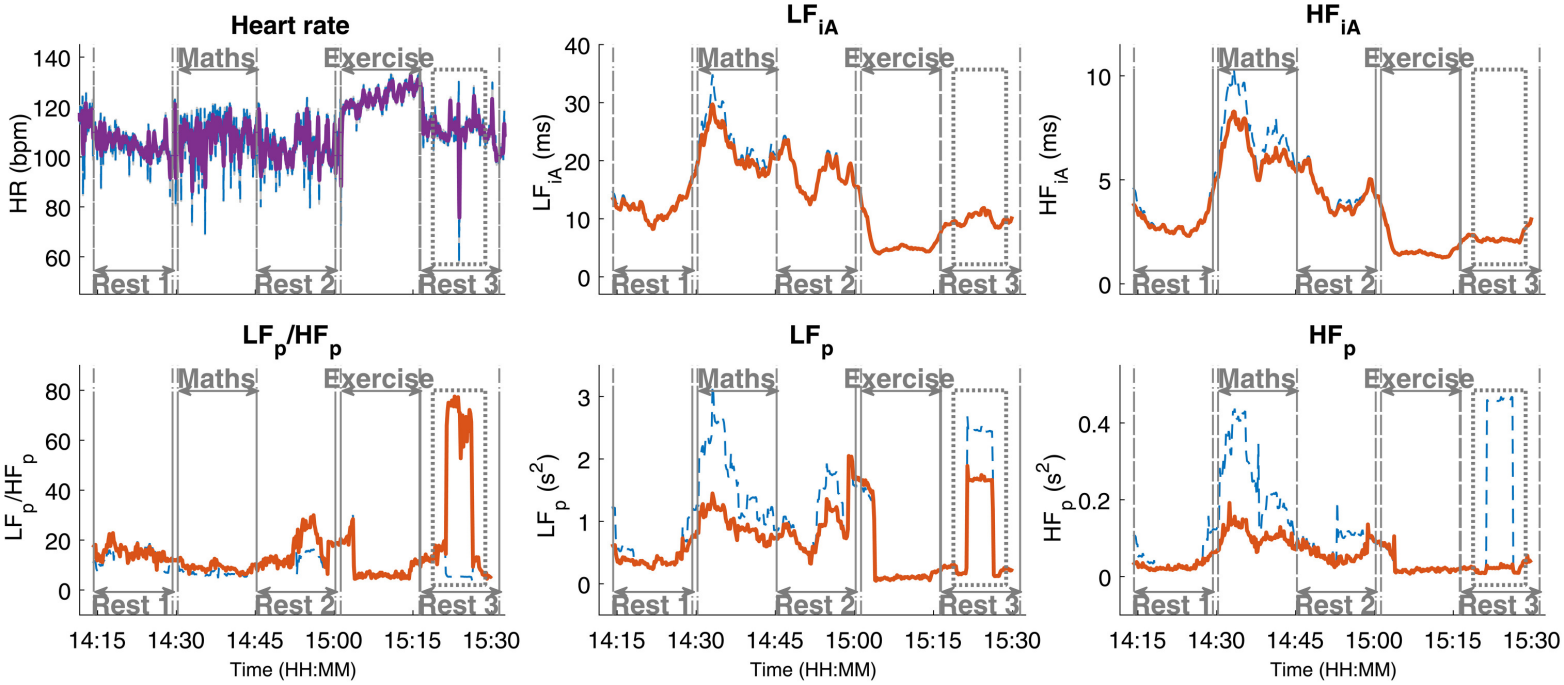
Instantaneous amplitude (iA) as a stress parameter. Observe its advantage over the low- (LF) and high-frequency (HF) power parameters, especially in the presence of artifacts of short duration and large amplitude, such as in the segment *Rest 3*: LF_iA_ and HF_iA_ are not affected by them. The results based on the raw NNI are designated in blue, and the results based on a preprocessed NNI time series, where sharp features have been removed, in orange.

Real-world recordings pose additional challenges and induce artifacts in the NNI time series, such as random and sudden jumps or abrupt changes in heart rate, which cannot be removed by standard bandpass filtering. Such events may therefore cause incorrect estimates of the LF_p_ and HF_p_ in the affected data segments. Furthermore, artifacts with a large amplitude, for example those produced by a deep breath, are reflected in approximately rectangular patterns (with a width corresponding to the length of the applied time window) in the plots for the LF_p_, HF_p_, and LF_p_/HF_p_ stress metrics over time, as indicated in Figure 3 in the segment *Rest 3*. The blue dashed lines in the panels for LF_p_, HF_p_, and LF_p_/HF_p_ denote the results using the raw NNI time series and the solid orange lines denote the results obtained after the removal of sharp peaks (see below). In order to mitigate the misleading influence of such events, while maintaining the full use of the information in the LF and HF frequency bands, we propose a new approach (outlined in **Algorithm 1**), whereby after bandpass filtering the HRV signal into the low- and high-frequency bands, the Hilbert transform is applied to both resulting time series in order to generate the corresponding complex analytic signals which exhibit time-varying amplitudes (Mandic et al., 2013; Looney et al., 2014; Hemakom et al., 2016), the so called instantaneous Amplitude (iA) concept (Looney et al., 2008). The so obtained signals are processed using the same time windows as those used for the other stress metrics, typically 5 min long sliding windows with a 10 s sliding increment (that is a 4 min 50 s overlap). The 20% largest and smallest signal values in every time window under consideration are removed and the mean amplitude value is calculated. This removes the outliers, while at the same time the essential information is retained: the average amplitude of a large proportion of the time window for both frequency bands, the instantaneous amplitude of the low frequency band (LF_iA_) and the instantaneous amplitude of the high frequency band (HF_iA_). For example, the segment *Rest 3* in Figure 3 (HR, top left) contains a short occurrence of anomalously low heart rates in the middle of the segment. This impedes the correct power estimation in the LF_p_ and HF_p_ frequency bands (lower middle and right panels) and yields a curve with a rectangular shape in the LF-power and HF-power panels (dashed blue line on the lower middle and right panels). The pre-conditioning of the NNI by removing sharp peaks from the NNI time series successfully removed the effect of this peak on the HF_p_ but not on the LF_p_ (the results after the peak removal are displayed using solid orange lines in all panels). Removing more components from the NNI in order to smoothen the LF_p_ would yield a larger difference between the blue and orange lines for LF_p_ and HF_p_, and would cause a loss of even more information in those stress metrics. For the LF_iA_ and HF_iA_, such pre-processing is not necessary, as exemplified by the absence of artifacts in the plots in Figure 3 (top middle and top right panels).

**Algorithm 1:** A procedure for using the instantaneous amplitude as a stress metric

1: Bandpass-filter the NNI into two bands, the LF and the HF
2: Apply the Hilbert transform to LF and HF to generate analytical signals
3: From the complex-valued analytic signals, obtain the amplitude at every point in time, the iA
4: Divide signals into time windows, typically sliding windows with a length of 5 min and a 10 s increment
5: In every time window, exclude the 20% largest and smallest values, to remove outliers
6: Calculate the mean for every time window

### 2.4. Normalization of the power bands

Standard one-dimensional analyses of LF_p_ and HF_p_ suggest that it is useful to investigate relative contributions of the individual bands to the total power [normalized LF_p_ (LF_n_) and normalized HF_p_ (HF_n_)] instead of, or in addition to, examining their absolute power values (Pagani et al., 1986). In theory, this would incorporate the state of the opposing nervous system into the analysis and would enable cross-subject comparisons by removing the influence of their individual baseline values. As a rule of thumb for a reliable estimation of the power in oscillatory data, the length of time windows should be at least ten times the period of the lowest frequency of interest. Therefore, for 5 min = 300 s time windows, reliable power estimation is only possible for oscillations with periods shorter than 300 s/10 = 30 s (or equivalently frequencies higher than 0.03 Hz). In addition, the timing of heart beats effectively defines the sampling rate of the original non-interpolated NNI time series, which in effect restricts the maximum frequency that can be estimated. Therefore, the Nyquist frequency corresponds to the half of the heart rate, meaning that for heart rates ≥60 bpm, the reliably quantifiable frequency range for 5 min time windows occupies the frequencies from 0.03 Hz (lowest frequency with 10 oscillations in 5 min) to 0.5 Hz (half of a heart rate of 60 bpm) (Heathers, 2014). For this frequency range, most of the total power (*TP*) of the NNI time series is observed in the LF and HF bands, and thus:

(2) $$LF_{p}+HF_{p}\approx TP$$

(3) $$LF_{n}=\frac{LF_{p}}{TP}\approx \frac{LF_{p}}{LF_{p}+HF_{p}}$$

(4) $$\begin{array}{l} HF_{n}=\frac{HF_{p}}{TP}\approx \frac{HF_{p}}{LF_{p}+HF_{p}}\\ \;\Rightarrow LF_{n}+HF_{n}\approx \frac{LF_{p}}{LF_{p}+HF_{p}}+\frac{HF_{p}}{LF_{p}+HF_{p}} \end{array}$$

(5) $$\Leftrightarrow LF_{n}+HF_{n}\approx \frac{LF_{p}+HF_{p}}{LF_{p}+HF_{p}}\;=\;1$$

(6) $$\Leftrightarrow HF_{n}\approx 1-LF_{n}$$

Observe from Equation (6) that the power normalization corresponds to projecting all combinations of LF_p_- and HF_p_-values onto a straight line in a 2D scatter plot, given by *HF*_n_ ≈ 1 − *LF*_n_. In this way, again one degree of freedom is lost, while Equation (6) indicates that the use of LF_n_ is almost equivalent to using either HF_n_ or LF_n_/HF_n_ alone (Heathers, 2014), as these are linearly related. Therefore, in the subsequent analysis we shall employ mainly absolute powers, while for reference the normalized powers are shown in tables in the Results section.

### 2.5. Categorization of stress states and statistical tools

The difference in HRV parameters in various practical scenarios is typically assessed through a statistical comparison of the distributions of stress parameters; when their means differ with a high probability, the corresponding change in the event is considered to have a significant impact on the HRV. As our goal is to determine the state of a person based on HRV metrics, being able to differentiate between different scenarios is here defined as being able to categorize a given scenario based on HRV-parameters. This is because, even when it is possible to show that the distributions of stress values for any two scenarios are different, an observed statistical difference between two scenarios (e.g., via a Wilcoxon rank sum tests) is not sufficient for successful categorization. For example, two normal distributions with slightly different means are statistically different, but—due to the overlap of the two distributions—when only a small number of data points is available, it is not possible to assign these points to either distribution with high certainty. In a graphical representation, when 1D or 2D HRV values in various scenarios are visually separable, a reliable categorization is guaranteed.

With this in mind, we shall use the categorization accuracy (CA) as a metric for a quantitative comparison of performance between standard 1D parameters and the proposed 2D scatter plots, i.e., based on the proportions of data points correctly or incorrectly assigned to the state of the participant. As a classifier, we employed a support vector machine (SVM) with a polynomial kernel with two inputs (features in low and high frequency bands). In the case of 1D parameters, the second input is set to a constant value in order to provide categorization in a single dimension. An SVM is an algorithm to discriminate between data sets that belong to different categories. When applied to the two-dimensional data in this study, this approach constructs regions with data points from various scenarios, whereby the margins between the boundaries of the so identified different regions and the data points closest to those boundaries are maximized. In this way, data points in a given area are—according to this model—most likely to arise from circumstances similar to the scenarios that were used to train the SVM. For a more detailed description of an SVM we refer to the Appendix in Supplementary Material. With a kernel SVM, all values in the 2D-space were able to be associated to a stress category.

The *p*-values of statistical differences between the scenarios in Part 1 are provided in the Results section. For each stress metric, Wilcoxon rank sum tests were employed on one average value per subject and scenario. Moreover, using Wilcoxon rank sum tests, the categorization accuracies of standard one-dimensional metrics are compared with the categorization accuracies of the proposed two-dimensional metrics.

### 2.6. Experimental setup

For a comprehensive verification of the proposed 2D analysis, two sets of recordings were performed. In the first scenario, ten subjects performed exactly the same task under the same circumstances, in order to compare traditional (normalized and non-normalized) power-related stress indices to the proposed approach which utilizes the instantaneous amplitude of the two frequency bands. Furthermore, this data set, enabled a quantitative comparison between the performances of the classifiers based on both one-dimensional HRV metrics and the suggested method in two dimensions. After quantitatively showing the advantages of the proposed methods on this larger data set, a range of different experiments were performed to conclusively demonstrate the possibility of categorizing the many types of stress, or in other words, to enrich Figure 2 with enhanced discriminative ability.

#### 2.6.1. Part 1: standardized protocol for mental and physical stress

While the interpretation of the two areas in Figure 2 (resting and moderately stressed) in the two-dimensional LF-HF representation is well understood, the association of a variety of possible physiological states to the full available span of LF-HF ranges remains a challenge, for example, when the LF and HF values are close to zero. To shed more light on the mapping of the whole spectrum of physical and mental states onto our 2D representation, especially when examining the difference between light physical and light mental stress, we designed an experiment which includes segments of mental and physical activities, together with resting periods for determining the baseline and recovery periods before, in between, and after the activities.

The protocol was as follows:

- 15 min: sitting, eyes closed
- 1 min: sitting, explanation of the next part
- 15 min: sitting, mental math
- 15 min: sitting, eyes closed
- 1 min: standing up, explanation of the next part
- 15 min: exercise on a stepper at a fixed rate
- 10 s: returning to the chairs
- 15 min: sitting, eyes closed

A total of five pairs of participants undertook the experiment, whereby in the mental math section the two participants in each pair competed against one another; one point was awarded for every correct response and one subtracted for every wrong answer. All trials were performed according to video instructions which dictated the duration of the intervals and the stepping frequency of the physical exercise (1 step per second), thus ensuring a uniform procedure across recording sessions and subjects. Furthermore, all trials started at the same time in the afternoon (between 14:05 and 14:20) within an 8-day period, the participants were instructed not to eat in the hour before the start of the experiment, and the temperature of the room was kept constant at 22°C. During the experiment, talking was only permitted during transition periods and to answer the mental math questions. The participants were male, between 23 and 38 years of age (mean: 28.6 years) and did not have any known health complaints. All participants were physically active, 9 participants had a body mass index (BMI) between 18.5 and 25 ($\hat{=}$ healthy weight) and 1 participant had a BMI between 25 and 30 ($\hat{=}$ overweight).

#### 2.6.2. Part 2: further applications

To further explore the benefits of the proposed analysis framework over established methods, in Part 2, the aim was to evaluate the proposed 2D analysis framework over a wide range of human activities, which included:

1. **Meditation**: The ECG of one participant during two sessions of Samatha meditation was recorded. The three stages were Pre Meditation, Sitting Meditation, and Post Meditation.
2. **Conference**: A subject was recorded while giving an oral presentation at a conference. The talk lasted for 20 min and further 30 min were recorded before and after the talk (Chanwimalueang et al., 2016).
3. **Pain**: A patient was recorded immediately before and during a varicose vein surgery that consisted of two parts, one comparably pain-free (Ablation) and the other more painful (Avulsion).
4. **Mental stress**: A subject underwent a protocol loosely based on the TSST, comprising different psychologically stressful events and resting periods (Kirschbaum et al., 1993), such as preparing for a presentation (10 min), giving a presentation (7 min), an arithmetic test (5 min), and a resting period (16 min).
5. **Emergency**: The heart rate of a cardiologist was recorded before, during and after the simulation of an emergency in a cardiology department.

## 3. Results

### 3.1. Part 1: standardized protocol for mental and physical stress

**Figure 4:**
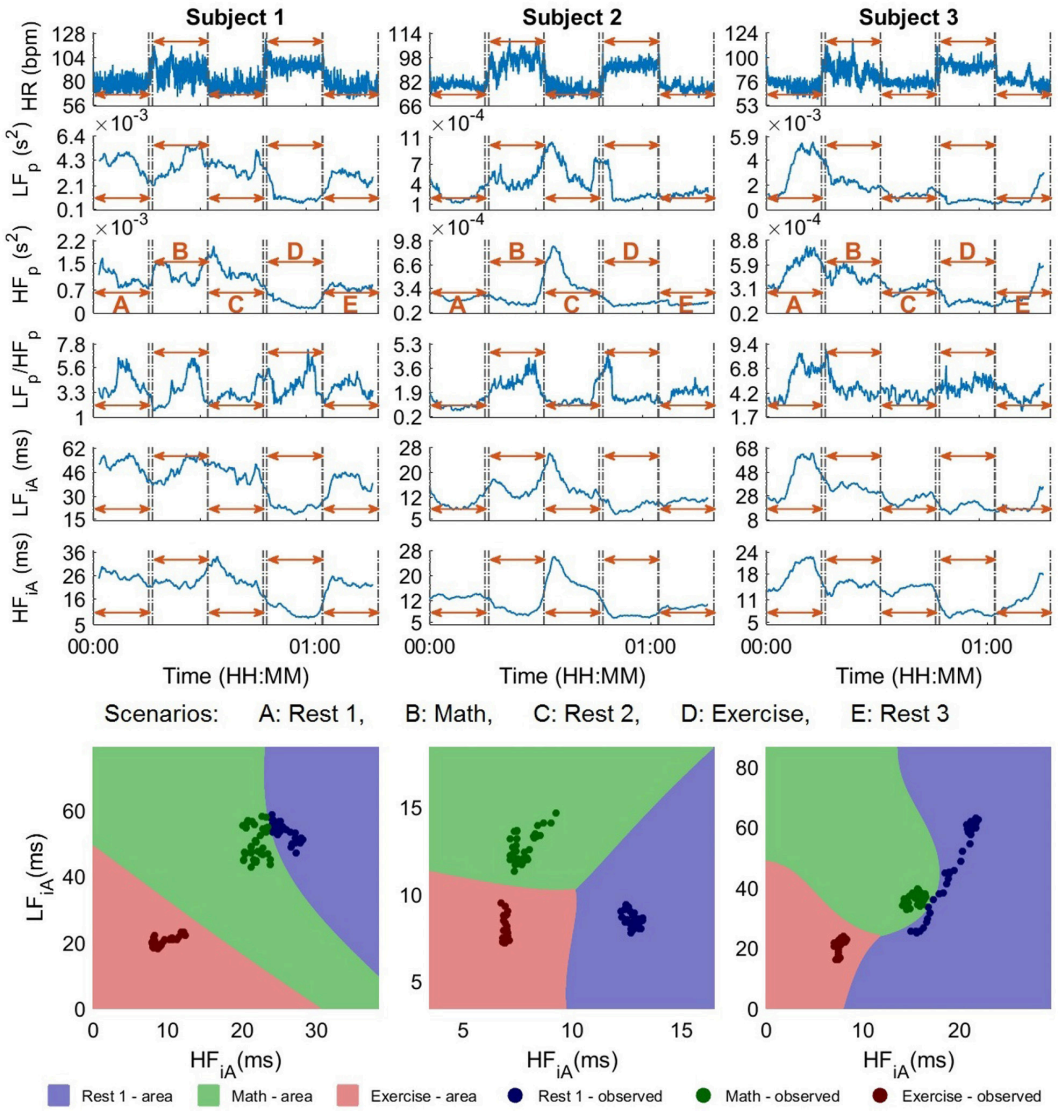
The standard univariate and the proposed bivariate stress parameters for 300 s sliding time windows, for the participants 1–3 in the experiments in Part 1. Upper: Stress parameters in 1D, that is, heart rate (HR), power in the low (LFp) and high frequency band (HFp), their ratio (LF_p_/HF_p_), the instantaneous amplitude in LF (LF_iA_), and the instantaneous amplitude in HF (HF_iA_). Lower: Proposed 2D stress parameters: LF_iA_-HF_iA_ scatter diagrams for the instantaneous amplitude (iA) of LF and HF, where the background color designates the partitioning into stress categories, achieved through the SVM classifiers. While it is not possible to distinguish between the stress scenarios using 1D parameters (upper), the separation using 2D representations (upper) was perfect.

In this experimental setup, the time windows were 300 s in duration, while in order to remove possible residuals from adjacent periods, buffer segments of 120 s were added at the start and the end of each scenario. The analysis was performed both qualitatively (visual inspection, see Figure 4) and quantitatively, with the results summarized in Table 1. As mentioned in Section 2.5, the goal of Part 1 was to establish the suitability of the proposed 2D analysis of HRV parameters to categorize different stress scenarios. The upper part of Figure 4 shows the evolution of 1D stress parameters over time, while the lower part displays the iA of the low and high frequency bands in a 2D scatter plot. The color-coded points indicate the observed states in the experiments and corresponding regions are color-coded in a similar shade.

**Table 1:** Categorization accuracy and statistical analysis for the scenarios *Rest 1, Math*, and *Exercise* across all ten subjects in Part 1.

**Metric**	**M1**	**M2**				**M3**
	**CA (%)**	**p_*R*−*M*_**	**p_*R*−*E*_**	**p_*M*−*E*_**	**CA (%)**	**CA (%)**
LF_p_	58.4	0.52	0.24	0.12	46.8	80.4
HF_p_	52.1	0.47	<0.01	0.06	59.5	88.6
LF_p_/HF_p_	48.8	0.16	0.16	1.00	52.1	79.9
2D_p_	**76.7**	nA	nA	nA	**75.7**	**98.3**
LF_n_	44.7	0.31	0.24	0.68	55.5	75.7
HF_n_	50.7	0.09	0.09	0.91	57.5	80.3
LF_n_/HF_n_	48.4	0.16	0.16	1.00	52.1	79.9
2D_n_	**61.7**	nA	nA	nA	**71.2**	**92.0**
LF_iA_	40.9	0.16	0.08	0.04	57.8	78.3
HF_iA_	57.7	0.14	<0.01	0.12	65.3	94.0
LF_iA_/HF_iA_	54.7	0.03	0.02	0.31	56.7	83.2
2D_iA_	**75.1**	nA	nA	nA	**85.9**	**99.6**

*Categorization based on the raw, absolute, stress parameters is given in column M1, while panel M2 shows the values after standardizing (for each subject separately) by subtracting the median values of the “baseline” segment Rest 1 from the whole recording (this improved cross-subject comparisons), and column M3 shows the values produced with the categorization performed for all subjects individually. The statistical differences were calculated for one-dimensional parameters and denoted as follows: p_R−M_: Rest 1 and Math, p_R−E_: Rest 1 and Exercise, p_M−E_: Math and Exercise, while the two-dimensional parameters are denoted with 2D_j_, j∈{p, n, iA}. The values in bold are the categorisation accuracies for the proposed 2D analysis*.

We should mention that for some subjects, it was possible to distinguish between different stress states based on conventional 1D stress parameters, however, in most scenarios, the analysis based on 1D stress parameters did not even outperform the raw heart rate, when used as an indicator. The difficulty with using only the heart rate (or any other 1D index) is the inability to distinguish between two stressful events which arise from very different circumstances but lead to similar heart rates (or respective stress values). For instance, the heart rates for Subjects *1* and *3* (Figure 4, upper) were almost identical during *Math* and *Exercise*, and thus indistinguishable by standard 1D analyses, however, the respective scatter plots (Figure 4, lower) permit a clear discrimination between the two states.

Next, we performed a statistical assessment of the discrimination ability of the considered stress features over the experimental scenarios through Wilcoxon rank sum tests of the distributions of values of all participants in *Rest 1, Math*, and *Exercise* (see Table 1, M2). For almost all comparisons, the *p*-values were smaller when considering the iA than when considering the power of the low- and high-frequency bands. Since the *p*-values are not designed to be a categorization parameter, as outlined in Section 2.5, the following analysis will focus on the categorization accuracy rather than on *p*-values.

**Figure 5:**
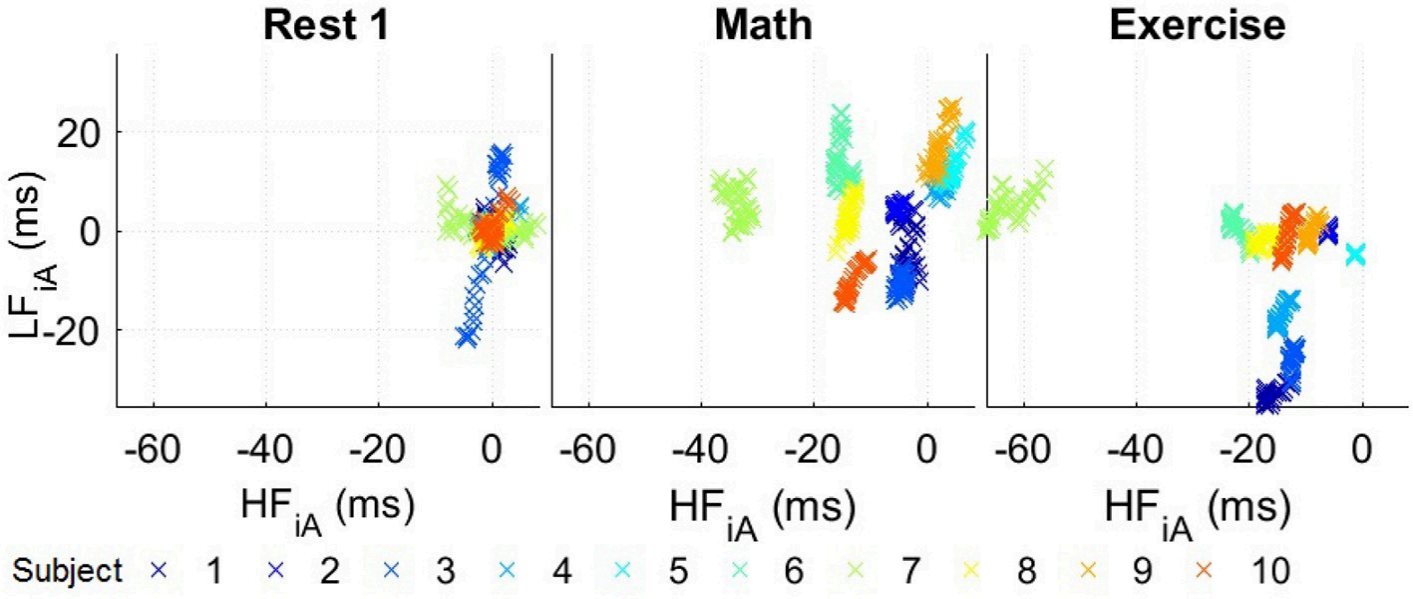
Standardized stress parameters in a 2D LF_iA_-HF_iA_ scatter diagram for all 10 participants in the experiments in Part 1, with 300 s time windows. The values for the instantaneous amplitude (iA) of the low (LF_iA_) and high frequency band (HF_iA_) of individual participants were standardized by subtracting the median values of *Rest 1*.

Compared to their corresponding resting segments, all subjects exhibited a lower HF_iA_ and almost all a lower LF_iA_ in the *Exercise* segment, but their absolute values varied between the participants. In order to address this problem and to be able to compare the reaction of different subjects to the same circumstances, the median value of stress parameters during the first resting period of each subject was subtracted from the respective values in the other segments. Figure 5 summarizes the responses of the subjects to different scenarios. Overall, a comparison of *Exercise* and *Rest 1* reveals a decrease in HF_iA_ for almost all subjects while LF_iA_ remained either at a similar level or decreased. The stress response to the *Math* segment was more varied across participants; in most cases, the LF_iA_ increased and the HF_iA_ decreased, compared to the initial resting period. This may be caused by different physiologies or different degrees of engagement/competitiveness. Similarly, the state of the participants in the resting period after *Math* can depend on the self-evaluation of their performance in the test. Those potential psychological variabilities did not exist for *Exercise*, though the last resting period may depend on the ability of the participants to recover from physical exercise.

After the standardization of the HRV values, the categorization accuracy improved, as displayed in Table 1 from panels M1 to M2 (85.9%). The accuracy for all 2D parameters was substantially higher than for their respective 1D counterparts. Furthermore, the best results were achieved using the proposed iA and, as expected, the performance of the normalized powers was the lowest among the three (absolute and normal powers and instantaneous amplitudes) in the 2D representation.

### 3.2. Part 2: further applications

**Figure 6:**
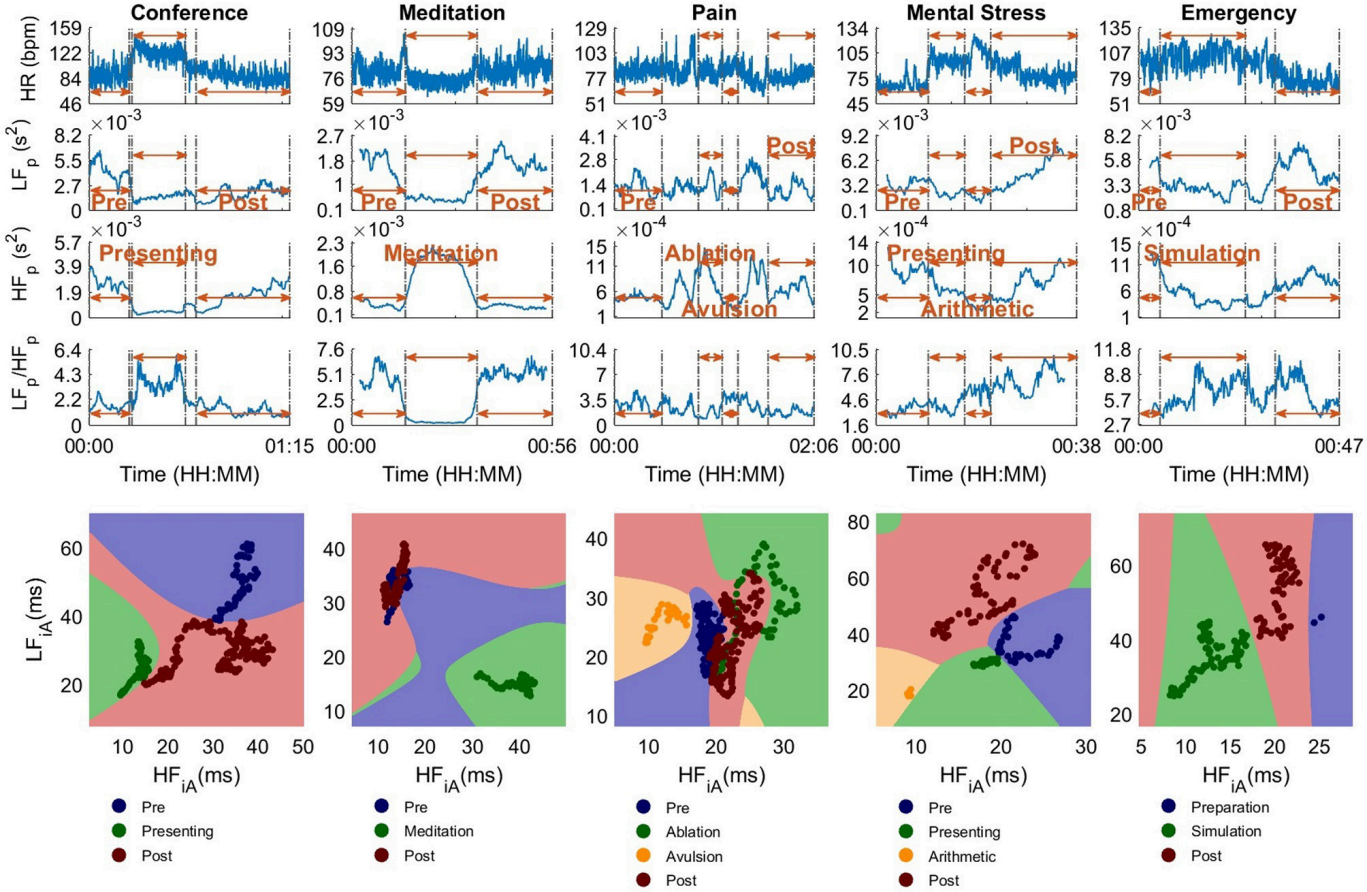
The standard univariate and the proposed bivariate stress parameters for 250 s sliding time windows, for experiments in Part 2. Upper: Stress parameters in 1D, that is, heart rate (HR), power in the low- (LF_p_) and high-frequency band (HF_p_), and their ratio (LF_p_/HF_p_). Lower: Proposed 2D stress parameters. The LF_iA_-HF_iA_ scatter diagrams of the instantaneous Amplitude (iA) in LF and HF, where the background color designates the partitioning into stress categories, achieved through the SVM classifiers. The scatter diagrams in the lower part are more suitable to differentiate between different scenarios than the 1D graphs in the upper part.

Figure 6 (upper) shows 1D stress parameters over time, calculated for the five experiments with different scenarios and subjects, while Figure 6 (lower) displays the corresponding color-coded 2D scatter plots. The results of the quantitative analysis are shown in Table 2 and for the analysis and figures in this section, 250 s time windows were used, as some scenarios lasted for less than 5 min.

**Table 2:** Categorization accuracy (CA) for the stress scenarios in the experiments in Part 2.

**Metric**	**Categorization accuracy (CA)(%)**				
	**Conference**	**Meditation**	**Pain**	**Mental stress**	**Emergency**
LF_p_	77.9	75.6	37.1	75.6	89.5
HF_p_	89.1	80.5	81.7	64.1	93.8
LF_p_/HF_p_	81.2	84.6	64.9	81.7	64.2
2D_p_	**98.5**	**82.9**	**86.1**	**99.2**	**98.8**
LF_n_	80.0	75.6	64.6	82.4	64.8
HF_n_	81.2	82.9	64.4	80.2	64.2
LF_n_/HF_n_	81.2	84.6	64.9	81.7	64.2
2D_n_	**88.2**	**84.6**	**66.3**	**93.1**	**82.1**
LF_iA_	77.9	75.6	50.7	84.0	89.5
HF_iA_	80.6	75.6	80.2	63.4	100.0
LF_iA_/HF_iA_	81.2	81.3	59.9	80.9	61.7
2D_iA_	**100.0**	**81.7**	**86.9**	**100.0**	**100.0**

*The values for Meditation are low because Pre and Post are practically the same states (see Figure 6). Otherwise, the accuracy for Meditation is 100.0% for all metrics. The values in bold are the categorisation accuracies for the proposed 2D analysis*.

For the *Conference* scenario, the value of LF_p_ during the *Presenting* stage differed considerably from the *Pre* period, but was similar between the *Presenting* and *Post* segments. The values of HF_p_ enabled a discrimination between *Presenting* and *Pre* and between *Presenting* and most of *Post*. When combining LF_p_ and HF_p_ into LF_p_/HF_p_, the beginning and end of *Presenting* exhibited a high LF_p_/HF_p_ value and the middle part had a similar amplitude to some peaks in the *Pre* and *Post* stages. Although no clear difference could be seen between *Pre* and *Post*, the proposed 2D scatter plots in Figure 6 (lower) were able to clearly distinguish between *Presenting* and the segments before and after. As a consequence, categorization accuracies based on the 2D parameters were considerably higher (CA(2D_iA_) = 100%) than the best accuracy for the 1D parameters (CA(HF_p_) = 89.1%). Overall, *Post* had lower LF_p_ and higher HF_p_ values, with the values with lower HF_p_ representing the time closest to finishing the presentation. This is not unexpected, since anxiety is likely to reduce after the presentation.

**Figure 7:**
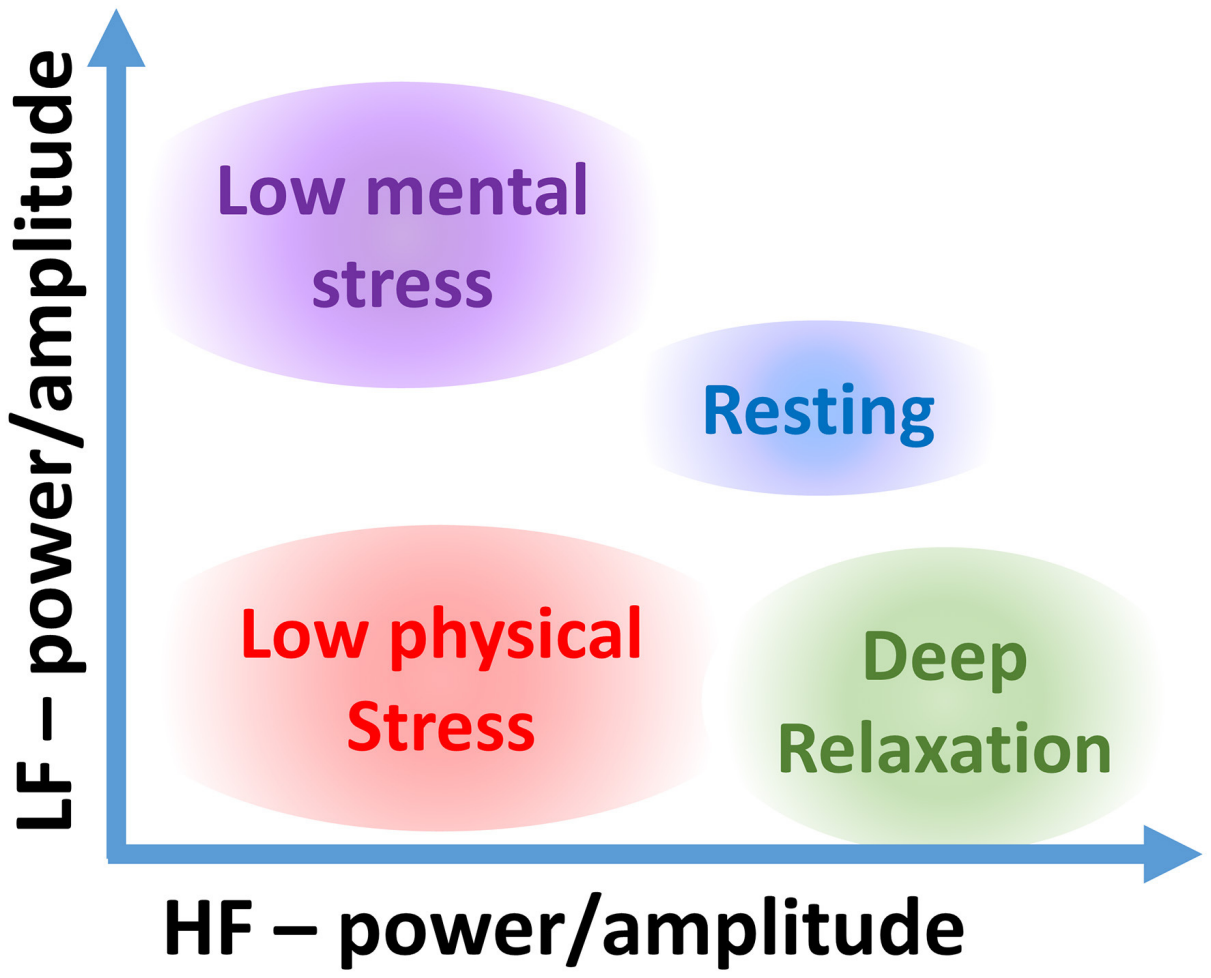
Stress categorization within the proposed two-dimensional LF-HF representation for various types of stress.

The participant in *Meditation* exhibited large differences between the *Meditation* stage and the periods before and after, for all stress parameters. The categorization accuracies were low since the stages *Pre* and *Post* are essentially the same. Based on this experiment, a state of very low physical exercise (sitting) and high relaxation (meditating) can be assigned to the upper left corner of the two-dimensional LF-HF scatter diagram in Figure 7.

In *Pain*, only the scatter plot of iA could distinguish, without ambiguity, between the painful part of the surgery (*Avulsion*) and the other stages. Furthermore, the overlap between *Ablation* and *Pre* was small in the scatter plot, while just one standard 1D feature, HF_p_, differed between these two scenarios.

Of all one-dimensional parameters in *Mental Stress* (see Experimental setup, Part 2, topic 4), only HF_p_ indicated an increase in the stress level (i.e., a reduced activity in HF_p_) until *Arithmetic*, and a decrease afterwards. The LF_p_/HF_p_ did not enable a differentiation between *Pre* and *Presentation* or *Arithmetic* and *Post*. However, when viewed in 2D, as desired, the overlaps between various scenarios were small, a crucial feature which is not possible to achieve with 1D metrics shown in Figure 6 (upper). Accordingly, the categorization accuracies for 2D_p_ and 2D_iA_ were at least 16% higher than the best performance for the respective 1D parameter (see Table 2).

During the simulation of a cardiac *Emergency*, LF_p_ and HF_p_ were different between the actual simulation and the stages before and after. Observe that the LF_p_/HF_p_ did not enable a differentiation between *Simulation* and *Post* based on 1D parameters, mainly because of an increase in LF_p_ during *Post*. In the 2D scatter plots, *Simulation* and *Post* could be clearly differentiated, with a categorization accuracy of 100.0%.

## 4. Discussion

Figures 4–6 and the results of the quantitative analyses in the respective tables (Tables 1, 2) indicate that:

1. In most cases the categorization based on the proposed iA performed similarly or better than based on the respective LF and HF powers, and
2. 2D scatter plots were superior to 1D analyses at distinguishing between different stress scenarios.

While in some cases a 1D stress metric achieved an accuracy similar to that of the 2D representation, the best performing 1D parameter varied across different experiments, so that the “universal” one could not be known in advance. In all cases, the most reliable and accurate results were obtained within the 2D analysis framework based on the instantaneous amplitude.

Overall, the results indicate that for more accurate characterizations of stress it is necessary to personalize the categorization of stress scenarios according to the participating individuals and their individual baselines. This makes it possible to assign different states of body and mind to the whole span of areas in the 2D scatter plots (see Figure 7). We would also like to highlight that for a more rigorous categorization of stress states it is necessary to consider non-overlapping test and training data sets. This, however, requires a specifically designed large-scale study, and is thus out of the scope of the current proof-of-concept study. It is reasonable to assume that a superior categorization on a training data set, as demonstrated here, will also lead to an enhanced categorization on the test data set, relative to the 1D counterpart metrics. While the latter statement does not hold in general, it is the case here, as it is mathematically not possible for the SVM to reduce its performance when considering two parameters instead of one. In case one of the inputs does not contain any meaningful important information, the SVM would categorize the data points without it by creating vertical or horizontal decision boundaries, or in case the ratio was the optimal parameter, the SVM would categorize along the diagonal in the 2D scatter diagrams. This all indicates the usefulness of the proposed 2D HRV parameters for stress categorization; our future work will aim to create personalized 2D stress metric for a larger cohort of participants.

Generally, it is assumed that the HF_p_ is low for mentally or physically stressful events and high for periods with low stress, and vice versa for the LF_p_. For the HF_p_, this was proven correct in all the considered experiments. However, the LF_p_ did not follow the expected behavior. In some very stressful situations (and in very painful ones—the participant in *Pain* gave the surgery a pain score of 7/10), such as *Presenting, Avulsion, Arithmetic*, and *Simulation*, the LF_p_ had the lowest values in the respective experiments.

In addition to the reported influence of the heart rate on HRV, as described in Billman (2013a) and Sacha (2014), in an attempt to explain the low LF_p_ values during physical exercise (confirming a similar result obtained by Arai et al., 1989) we focus on the ANS activity and its influence on the HRV. Recall that, instead of the total mean level of the activity of PNS and SNS, the HRV only reflects its variation, that is the fluctuation around its mean level (Malik et al., 1996), and not the absolute activity level. Our conjecture is that during instances of high sympathetic tone, the concentration of stress messengers (neurotransmitters and hormones) might be close to saturation, leading to a reduction in the LF and HF band magnitudes, a subject of a future study. This finding further highlights the importance of considering LF and HF jointly (e.g., within a scatter diagram) rather than through univariate LF/HF measures, as a low LF_p_/HF_p_ due to a low LF_p_ has a completely different meaning from a low LF_p_/HF_p_ due to a high HF_p_, i.e., physical exercise vs. a state of relaxation (see *Meditation*).

## Conclusion

This study has demonstrated that a simultaneous consideration of the activity of the low frequency band (LF) and high frequency band (HF) in heart rate variability (HRV) through 2D scatter diagrams, instead of standard univariate metrics such as the LF/HF ratio, significantly improves the discrimination ability in the analysis of physical and mental stress. The additional degree of freedom, obtained through 2D scatter diagrams, has not only made it possible to accurately discriminate between stress states, but also to make unnecessary the restrictive assumption of a linear relationships between the activity of the nervous systems and the power of the frequency bands, or a reciprocal interaction between the power in the low frequency band (LF_p_) and power in the high frequency band (HF_p_). Comprehensive analyses over a range of real-world scenarios have demonstrated that the proposed framework provides statistically significant discrimination across all subjects, and over various stress scenarios and resting periods. Future work will consider the personalization of the 2D diagrams in order to more accurately categorize the stress of an individual.

The proposed 2D analysis framework, together with the considered real-world and lab-based scenarios, has allowed us to enhance the discrimination ability in the stress categorization diagram. Note that the considered experiments did not include a case where the same subject undergoes high mental stress (e.g., a conference presentation) and low physical stress; this will be examined in a follow-up study.

## Ethics statement

This study was carried out in accordance with the recommendations of the Joint Research Office at Imperial College London, reference ICREC_12_1_1, extended in December 2013, with written informed consent from all subjects. All subjects gave written informed consent in accordance with the Declaration of Helsinki. The protocol was approved by the Joint Research Office at Imperial College London.

## Author contributions

WvR, TC, TA, UJ, VG, DM designed research; VG built the acquisition device; WvR, TC, TA performed research and analyzed data; WvR, TC, TA, UJ, DM wrote the paper.

### Conflict of interest statement

The authors declare that the research was conducted in the absence of any commercial or financial relationships that could be construed as a potential conflict of interest.
