# Supplementary material for: Resolving Ambiguities in the LF/HF Ratio: LF-HF Scatter Plots for the Categorization of Mental and Physical Stress from HRV
Source: Front Physiol. 2017 Jun 14;8:360. doi: 10.3389/fphys.2017.00360 (PMC5469891; doi:10.3389/fphys.2017.00360)
Supplement: Supplementary file 1 [file Appendix.pdf]

SUPPLEMENTARY FIGURE

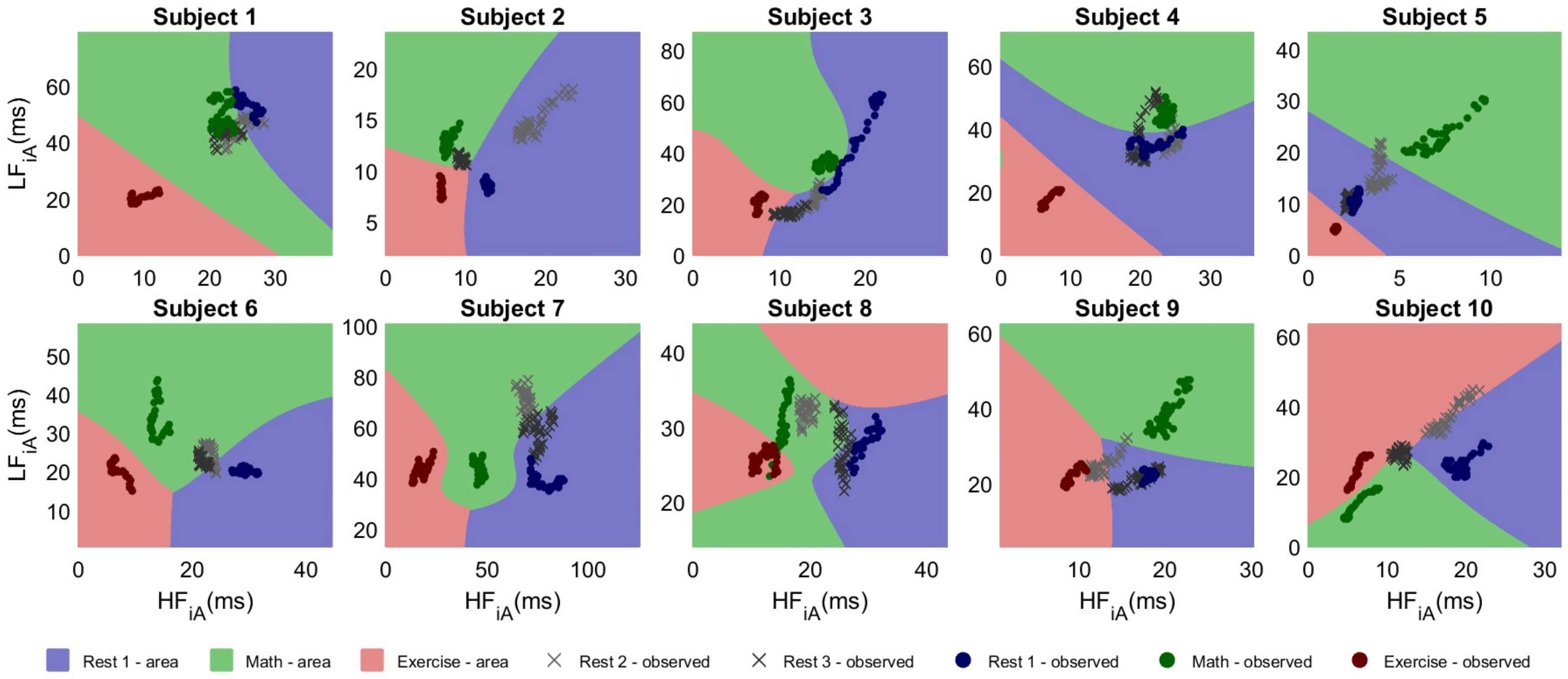

Figure S1. The proposed bivariate stress parameters LF_{iA} and HF_{iA} for 300 s sliding time windows, for all 10 participants in the experiments in Part 1. The background color designates the partitioning into stress categories, achieved through the support vector machine (SVM) classifiers. The resting/recovery periods after the tasks, Rest 2 and Rest 3, are denoted with gray 'x', e.e. in the blue region for Subject 2.

SUPPORT VECTOR MACHINES

The aim of an SVM is to find a geometrical boundary between two classes of data in order to separate these classes in one optimal way, its output contains binary values $y_i \in \{+1, -1\}$, independent of the nature of the considered data set; $y_i = +1$ for Class 1 and $y_i = -1$ for Class 2. For a given classification task, as shown in Fig. S2, multiple (hyper-)planes (in this particular case a solid straight line) can form such a boundary between the samples $\mathbf{x}_i$ (i=1,2,...,M) belonging to Class 1 and Class 2. To achieve the best possible decision boundary, the optimisation task for an SVM amounts to finding the hyperplane which yields the maximum class separation margin. In the example in Fig. S2, data points have two coordinates, x_1 and x_2, and the hyperplane between the two classes can be described by:

$$\mathbf{w}^T\mathbf{x} + b = 0, \tag{1}$$

where $\mathbf{x}$ is a point in the considered 2D-space, $\mathbf{w}$ the weight vector to be found, and b a bias term. A given $\mathbf{x}_i$ is assigned to Class 1 for $\mathbf{w}^T\mathbf{x}_i + b \geq 0$, and to Class 2 for $\mathbf{w}^T\mathbf{x}_i + b < 0$. The *support vectors* are a subset of vectors taken from the data, $\mathbf{x}_i$, in the original data set which are positioned on the margin boundaries, designated by broken lines on both sides of the optimal hyperplane. For non-separable datasets, there will be some overlap between the classes, and *margin errors* or *slack variables* ξ_i are introduced, which describe the distance between a data point $\mathbf{x}_i$ and its corresponding class (e.g. a misplaced blue circle in Class 2 in Fig. S2). The optimal separation hyperplane is then found by minimizing the following cost function:

$$\min_{\mathbf{w}} J(\mathbf{w}, \mathrm{b}, \boldsymbol{\xi}) = \frac{1}{2}\mathbf{w}^T\mathbf{w} + C\sum_{i=1}^{M}\xi_i, \tag{2}$$

subject to

$$y_m[\mathbf{w}^T\mathbf{x}_i + b] \geq 1 - \xi_i, \quad \xi_i \geq 0, \quad i = 1, 2, ..., M, \tag{3}$$

where $\boldsymbol{\xi} = [\xi_1, \xi_2, ..., \xi_M]^T$ and C is a user-defined parameter. Therefore, if $\mathbf{x}_i$ is on the incorrect side of the hyperplane, then $\xi_i > 1$, while for an $\mathbf{x}_i$ on the correct side of the hyperplane and within the margin, $0 < \xi_i < 1$, and $\xi_i = 0$ for $\mathbf{x}_i$ on the correct side of the hyperplane and outside the margin.

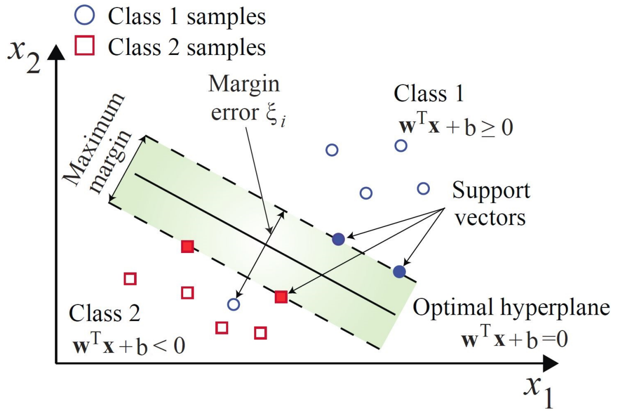

Figure S2. Principle of an SVM - the aim is to create an optimal hyperplane that maximises the margin between the two classes, Class 1 and Class 2.

For non-linear separation, as is the case in our study, *kernels* are introduced to transform the data set from the original linearly non-separable *input space* to a linearly separable *feature space*. For more detail regarding the principle of an SVM, refer to Cortes and Vapnik (1995), Haykin et al. (2009), Cichocki et al. (2017), Tobar et al. (2014).

REFERENCES

Cichocki, A., Lee, N., Oseledets, I., Phan, A., Zhao, Q., and Mandic, D. P. (2017). Tensor decompositions for dimensionality reduction and large-scale optimization. part 2: Applications and future perspectives. *Foundations and Trends® in Machine Learning* 9, 431–673. doi:10.1561/2200000059

Cortes, C. and Vapnik, V. (1995). Support-vector networks. *Machine learning* 20, 273–297

Haykin, S. S., Haykin, S. S., Haykin, S. S., and Haykin, S. S. (2009). *Neural networks and learning machines*, vol. 3 (Pearson Upper Saddle River, NJ, USA:)

Tobar, F. A., Kung, S., and Mandic, D. P. (2014). Multikernel least mean square algorithm. *IEEE Transactions on Neural Networks and Learning Systems* 25, 265–277. doi:10.1109/TNNLS.2013.2272594
